# The Uncoordinated-5 Homolog B Receptor Affects Hepatic Ischemia Reperfusion Injury

**DOI:** 10.1371/journal.pone.0041085

**Published:** 2012-07-25

**Authors:** Klemens König, David Köhler, Tiago Granja, Carla Jennewein, Nguyen Tran, Valbona Mirakaj, Friedemann Kröhnert, Peter Rosenberger

**Affiliations:** 1 Department of Anaesthesiology and Intensive Care Medicine, Tübingen University Hospital, Eberhard-Karls University Tübingen, Tübingen, Germany; 2 Clinic of Anaesthesiology, Intensive Care Medicine and Pain Therapy, University Hospital Frankfurt am Main, Johann Wolfgang Goethe University, Frankfurt am Main, Germany; 3 Department of Anaesthesiology, Perioperative, and Pain Medicine, Center for Experimental Therapeutics and Reperfusion Injury, Brigham and Women's Hospital Harvard Medical School, Boston, Massachusetts, United States of America; University of Colorado Denver, United States of America

## Abstract

Recent evidence has demonstrated additional roles for the neuronal guidance protein receptor UNC5B outside the nervous system. Given the fact that ischemia reperfusion injury (IRI) of the liver is a common source of liver dysfunction and the role of UNC5B during an acute inflammatory response we investigated the role of UNC5B on acute hepatic IRI. We report here that UNC5B^+/−^ mice display reduced hepatic IRI and neutrophil (PMN) infiltration compared to WT controls. This correlated with serum levels of lactate dehydrogenase (LDH), aspartate- (AST) and alanine- (ALT) aminotransferase, the presence of PMN within ischemic hepatic tissue, and serum levels of inflammatory cytokines. Moreover, injection of an anti-UNC5B antibody resulted in a significant reduction of hepatic IR injury. This was associated with reduced parameters of liver injury (LDH, ALT, AST) and accumulation of PMN within the injured hepatic tissue. In conclusion our studies demonstrate a significant role for UNC5B in the development of hepatic IRI and identified UNC5B as a potential drug target to prevent liver dysfunction in the future.

## Introduction

During several clinical conditions such as liver resection, liver transplantation or generalized shock, ischemia-reperfusion injury (IRI) is a common cause of liver dysfunction and hepatic failure [1]. Moreover hepatic IRI can contribute to the systemic inflammatory response syndrome with subsequent multiple organ failure [2]. Various underlying mechanisms of hepatic IRI are well known, including the induction of tissue necrosis, the generation of reactive oxygen species [3], the release of proinflammatory cytokines and the recruitment and activation of immunocompetent cells [4]–[6]. Notably, neutrophils play a key role in the development of hepatic IRI [4], [5]. Accumulation of neutrophils in the liver significantly contributes to hepathocyte necrosis by release of proteolytic enzymes and superoxide anions during hepatic IRI.

It is well established that activation and migration of leukocytes is controlled through the chemokine system [7]. However recent studies provide evidence that neuronal guidance proteins (NGP) and their receptors display an alternative class of guidance cues in the immune system that steer immune responses particularly with regard to activation and migration of leukocytes [8], [9]. NGP were first identified in the developing central nervous system (CNS), where neurons and axons are precisely guided to their final location by a balance of chemoattractive and chemorepulsive signals to establish the elaborate neuronal circuitry [10], [11]. Several families of such conserved neuronal guidance cues influencing axonal migration were identified to date. Recent data provide evidence that the NGP receptor Uncoordinated-5 homolog B (UNC5B) holds additional function outside the nervous system especially in the control of the immune system [12]–[16]. The NGP netrin-1, as endogenous ligand of UNC5B, has shown potent anti-inflammatory properties in animal models of hypoxia, ventilator associated lung injury, peritonitis and renal ischemia-reperfusion injury [17]. Tadagavadi et al. showed that netrin-1 application protected against renal IRI. This effect was not present after pre-treatment with an anti UNC5B antibody, suggesting that the protective effects of netrin-1 were mediated through an interaction with UNC5B.

Given clinical significance of hepatic IRI and the role of UNC5B for the control of an immune response, we pursued its role during hepatic IRI. For this we investigated the role of the UNC5B receptor in a murine model of hepatic IRI. We show here that UNC5B^+/−^ mice present with reduced hepatic IRI. Moreover functional inhibition of the UNC5B receptor with an UNC5B antibody dampened the extent of hepatic IRI.

## Methods

### Mice

Heterozygous UNC5B^+/−^ receptor knockout mice were kindly provided by Dr. Tessier-Lavigne. UNC5B^+/−^ mice were generated, validated and characterized as described previously [18]. UNC5B^+/−^ mice and controls were selected to be similar in age (6–8 weeks), weight (20–25 g) and randomised in gender. All animal experiments in this study are specifically approved by the Regierungspräsidium Tübingen and the local ethics committee of the Tübingen University Hospital.

**Figure 1:**
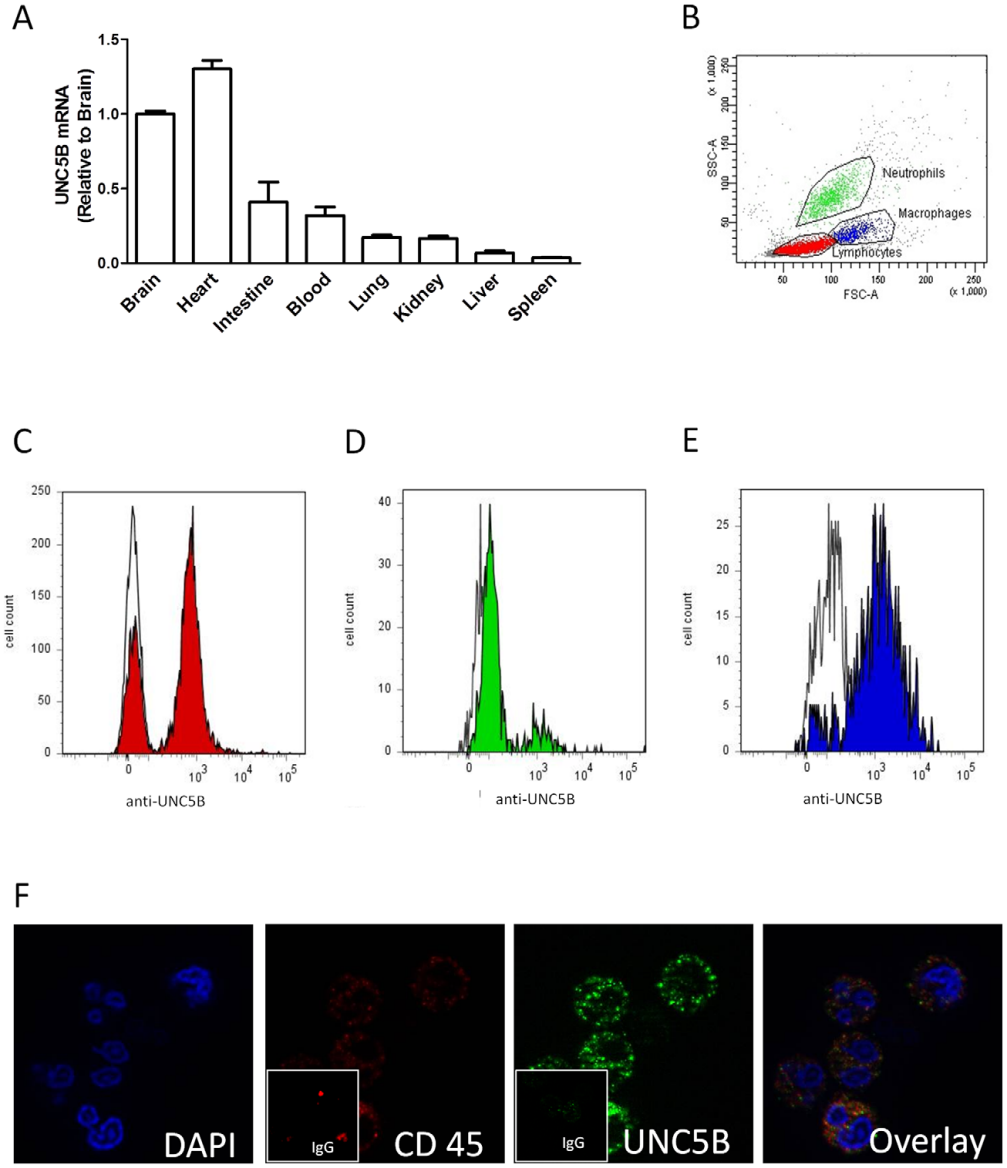
UNC5B expression outside the CNS. A) Relative UNC5B mRNA expression in brain, heart, intestine, blood, lung, kidney, liver and spleen in C57Bl/6 mice. B)–E) Flowcytometry of murine leukocytes gated and stained for the leukocyte marker CD45 (PerCP labeled), the macrophage marker F4/80 (APC labeled) and UNC5B (PE labeled). F) Immune fluorescence staining of human PMN was performed using anti-UNC5B and CD45 antibodies as primary and Alexa488-conjugated and CruzFluor™ 594-conjugated as secondary antibodies were used as well as isotype matched control antibodies. (Data are mean ± SEM, n = 4 per condition).

**Figure 2:**
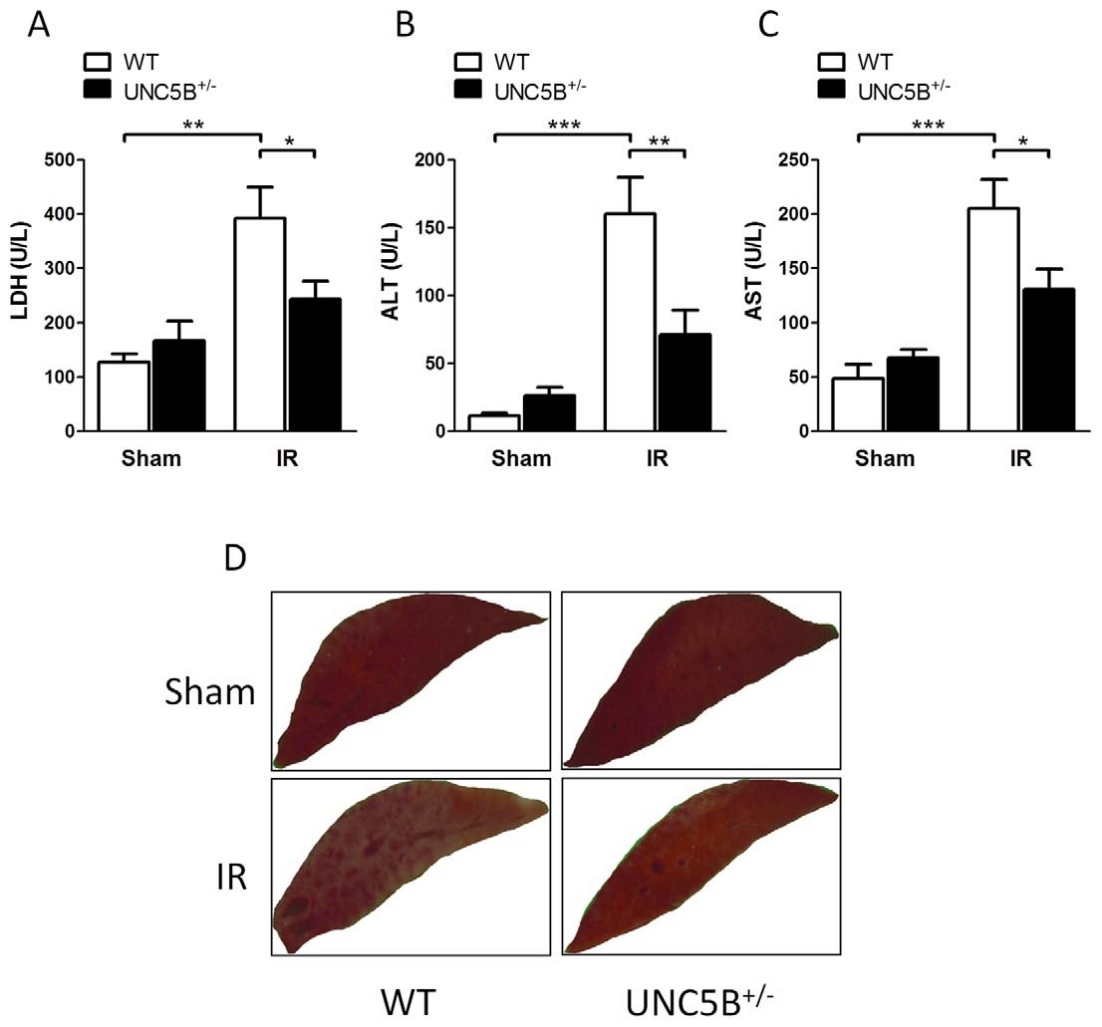
UNC5B^+/−^ animals demonstrate reduced hepatic IRI. WT and UNC5B^+/−^ animals underwent 30 minutes ischemia and 3 hours of reperfusion (IR) or sham operation before samples were taken. Serum was analysed for A) LDH, B) ALT, C) AST (Data are mean ± SEM, n = 6 per condition). D) Representative pictures of TTC stained liver lobes are shown (n = 4 per condition).

**Figure 3:**
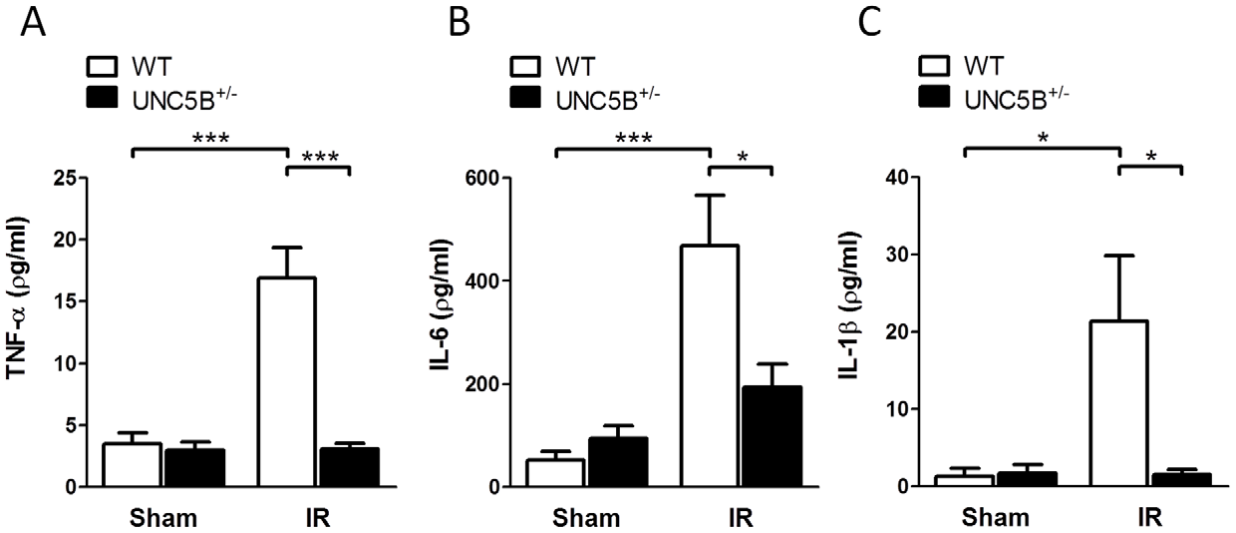
UNC5B^+/−^ animals display lowered systemic cytokine levels following hepatic IRI. Serum analysis of A) TNF-α, B) IL-6 and C) IL-1β of WT and UNC5B^+/−^ mice following IRI and sham operation are shown. Data are mean ± SEM, n = 6 per group.

**Figure 4:**
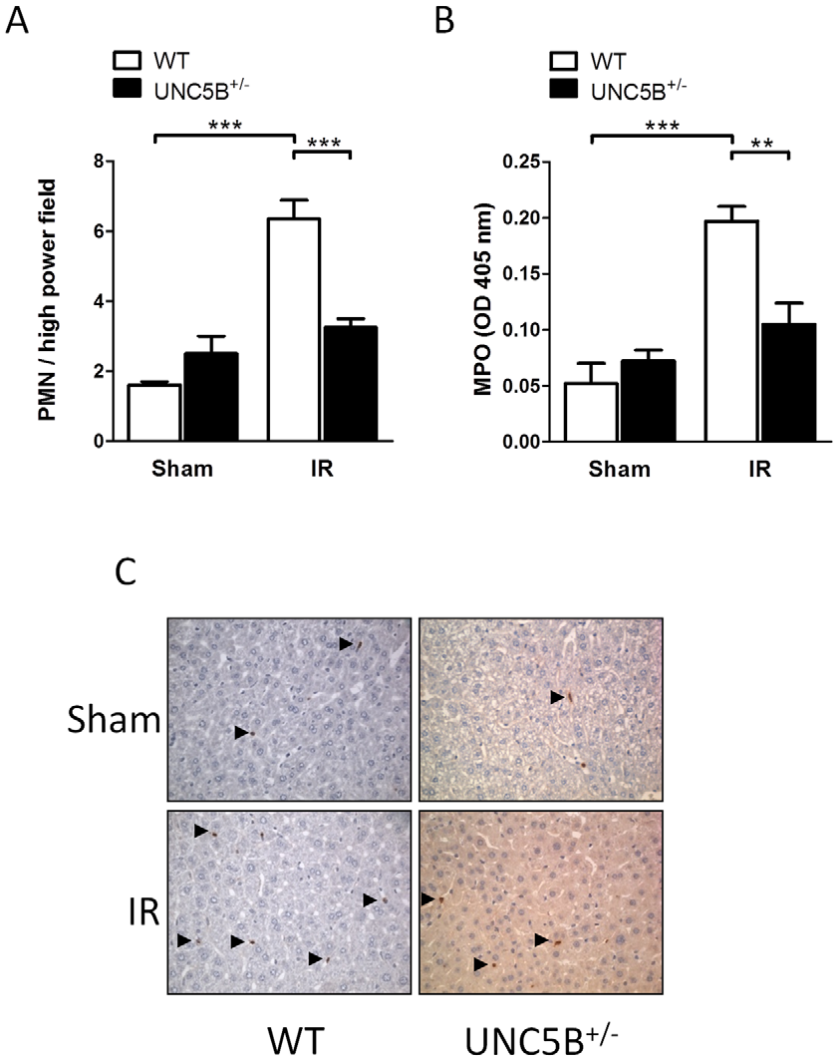
UNC5B^+/−^ animals show reduced PMN infiltration and activity following hepatic IRI. A) PNM counts per high power field in WT and UNC5B^+/−^ mice in the stained liver tissue. B) Relative MPO activity in hepatic tissue of WT and UNC5B^+/−^ animals C) Representative pictures of PMN staining of liver lobes (brown, marked with arrowheads) of WT and UNC5B^+/−^ mice after IRI and sham operation are shown (magnification 400×). Data are mean ± SEM, n = 6 per group.

**Figure 5:**
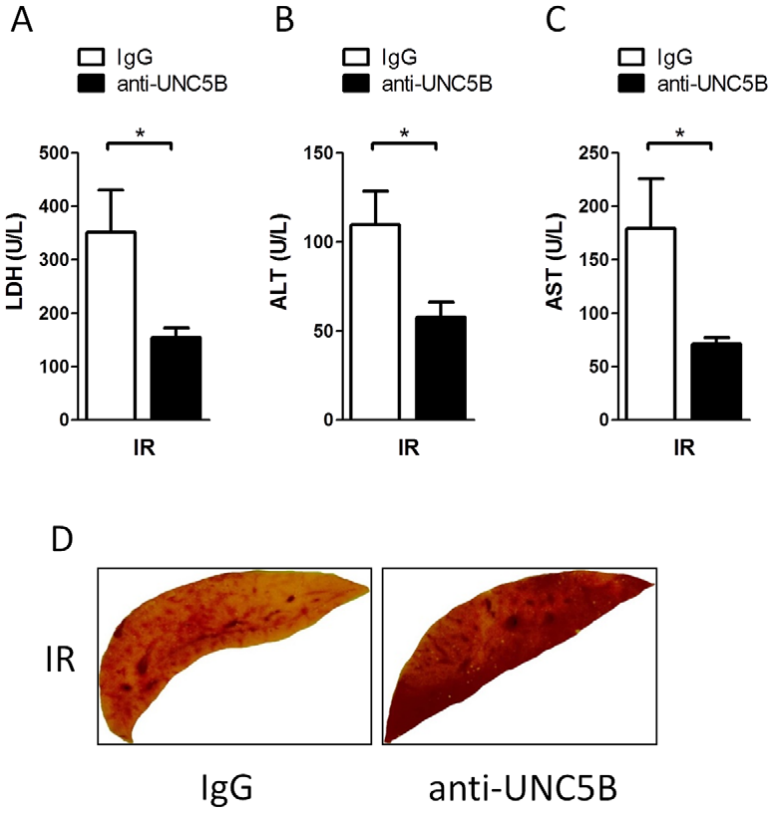
Administration of an anti-UNC5B antibody reduces hepatic IRI. WT animals treated with an anti-UNC5B antibody or Immunoglobulin (IgG) prior to the induction of IRI. A) LDH, B) ALT, C) AST in the serum was analysed (Data are mean ± SEM, n = 6 per condition). D) Representative pictures of TTC stained liver lobes are shown (n = 4 per condition).

**Figure 6:**
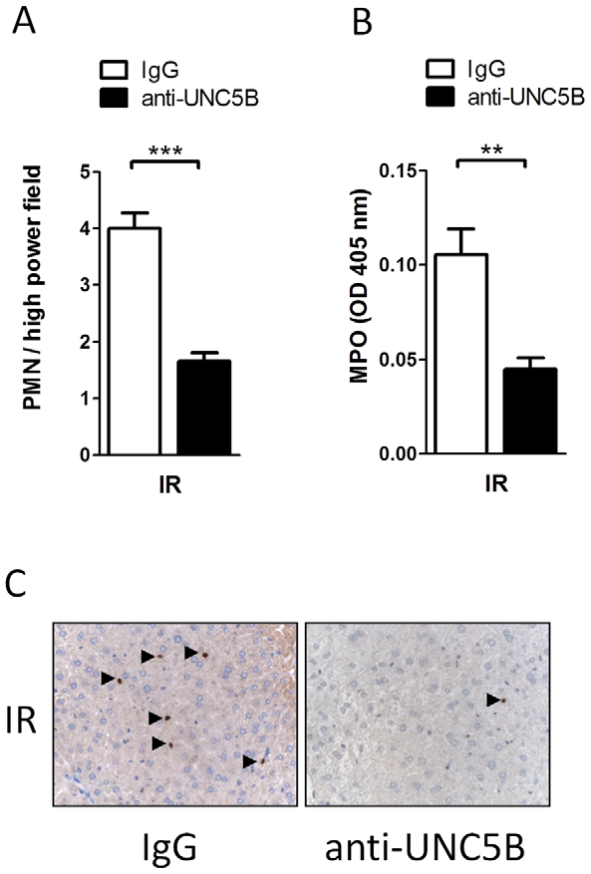
Anti-UNC5B antibody treatment diminishes hepatic PMN infiltration following hepatic IRI. A) PNM counts per high power field in the stained liver tissue. B) Relative hepatic MPO activity in mice pre-treated wit anti-UNC5B or IgG control. C) Representative pictures of histological PMN staining of liver lobes (brown, marked with arrowheads) of WT mice pre-treated with anti-UNC5B or IgG after IRI, are shown (magnification 400×). Data are mean ± SEM, n = 6 per group.

**Figure 7:**
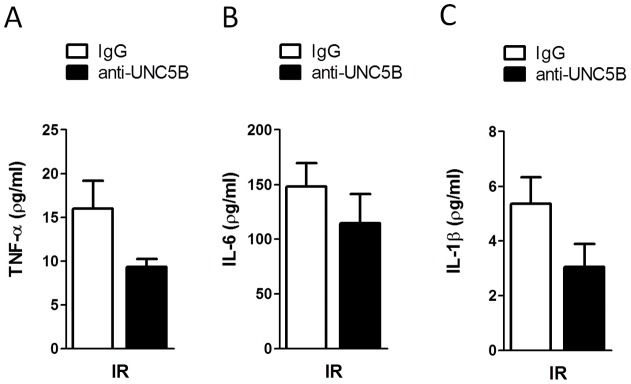
Functional inhibition of UNC5B trends to reduced systemic cytokine response after IRI. Serum of WT animals treated with anti-UNC5B antibody or Immunoglobulin (IgG) analysed for A) TNF-α, B) IL-6 and C) IL-1β after IRI. Data are mean ± SEM, n = 6 per group.

### Murine model of hepatic ischemia reperfusion injury

Following the induction of anaesthesia mice were placed on a heated table to maintain body temperature at 37°C. Midline laparotomy with caudal mobilisation of the intestine was performed to display the liver and the portal triad. Partial liver ischemia was induced for 30 minutes by reversible occlusion of the portal triad using a hanging-weight system as described previously [19]. This was followed by a reperfusion phase of 3 hours before animals were sacrificed and organs were harvested.

### Quantification of the liver injury

Serum alanine- (ALT) and aspartate- (AST) aminotransferase levels were measured with a colorimetric kit (Teco Diagnostics, Anaheim, CA) as described previously [19]. Lactate dehydrogenase (LDH) activity in the serum was measured using a photometric kit (Randox, Cumlin, UK).

### Antibodies used

Functional inhibition of UNC5B was performed by i.v. injection of anti-UNC5B antibody [17] (5 µg/mouse, R&D Systems, # AF1006) 15 minutes prior to surgical procedures. Appropriate isotype IgG was used as control.

### Real time PCR

Real-time RT-PCR was performed by a StepOnePlus™ Real-time PCR system (Applied Biosystems, Foster City, CA) to detect UNC5B, TNF-α, IL-6, CXCL1 and CXCL2 mRNA levels using the following primers. UNC5B sense primer 5′-GGACCTCCT TCAGTGCTACA-3′ and 5′-GCTAAGTCCTCATCCT-3′ anti sense primer, TNF-α sense primer 5′-ACTCCAGGCGGTGCCTATGT-3′ and 5′-TCCAGCTGCTCCTCCACTTG-3′ anti sense primer, IL-6 sense primer 5′- ACCGCTATGAAGTTCCTCTC-3′ and 5′-CTCTCCGGACTTGTGAAGTA-3′ anti sense primer, CXCL1 sense primer 5′-TAGGAACCCCCTCCTCAGCCCA-3′ and 5′-AAGTCCGAACTCCCGGTGTGGT-3′ anti sense primer, CXCL2 sense primer 5′-TGGGGAAGGACATCCCAGGGTC-3′ and 5′-ACAGAGCCCCGCCCCTAAGC-3′ anti sense primer. Samples were normalized with β-actin using sense 5′-GGTGGCTTTTAGGATGGCAAG-3′ and antisense 5′-ACTGGAACG GTGAAGGTGACAG-3′ primers.

### Cytokine measurement

Serum levels of IL-1β, IL-6 and TNF-α, were measured using a multiplex bead immunoassay kit (invitrogen™) in combination with a Luminex 100 system (Luminex corp., Austin, TX).

### Histological assessment

Immunohistochemical staining of PMN was performed from paraffin embedded tissue sections as described previously [20]. As primary antibody a rat anti mouse Ly-6b.2 (AbD Serotec, Düsseldorf, Germany) and as secondary antibody a biotinylated rabbit anti rat antibody (Vector Laboratories, Burlingame, CA) was used. Isolated human PMN from peripheral blood were stained as described previously [20]. Goat anti-UNC5B (Santa Cruz Biotechnology) and mouse anti-human CD45 (Abcam, clone: 0.N.125) antibodies as primary and Alexa488-conjugated donkey anti-goat and CruzFluor™ 594-conjugated donkey anti-mouse (Santa Cruz Biotechnology) as secondary antibodies were used.

### FACS analysis

Collected heparinised mouse blood was incubated with erythrocyte lysing solution for 5 minutes at room temperature. Cells were washed and incubated with an PE labeled UNC5B antibody (Abcam, Cambridge, UK, #ab54430), APC labeled F4/80 (eBioscience, San Diego, CA, #17480182), PerCp labeled CD45 antibody (BD Biosciences, Heidelberg, Germany, #557235) or isotype matched control antibody lightproof for 1 hour at room temperature. A BD FACS Canto™ II flowcytometer was used to perform FACS analysis employing BD FACS Diva™ software.

### Triphenyltetrazolium chloride staining (TTC)

Median liver lobes were extracted, placed on parafilm, frozen at −20°C for 30 min and cut into 1 mm slices. The slices were incubated with 1% TTC at 37°C for 30 min and fixed in 10% formalin.

### Data analysis

All data are presented as mean ± SEM. Statistical analysis was performed with GraphPad 5.0 software (GraphPad, San Diego, CA). For Multiple comparisons one-way ANOVA with Bonferroni adjustment was performed and for single comparison the unpaired Student's *t* test was applied. A value of P <0,05 was considered significant.

## Results

### Expression pattern of UNC5B

To validate our model we first addressed the question whether UNC5B is expressed in organs of C57Bl/6 mice outside the CNS. Real time PCR showed that UNC5B mRNA is expressed within several murine organs (Figure 1A). To specify the UNC5B expression on immunocompetent cells we performed flowcytometry using CD45 as a general marker for leukocytes and F4/80 to label macrophages. We found a robust signal for UNC5B on neutrophil, macrophage and lymphocyte populations (Figure 1B–E). This was confirmed by immunofluorescence staining of leukocytes (Figure 1F).

### Hepatic ischemia-reperfusion injury is reduced in UNC5B^+/−^ mice

To clarify the impact of UNC5B during hepatic IRI we used previously characterised UNC5B^+/−^ mice and C57BL/6 wild type (WT) mice. Following hepatic IRI, UNC5B^+/−^ mice displayed significant reduced serum levels of LDH, ALT and AST compared to WT animals (Figure 2A–C). TTC staining of injured liver lobes further exemplified reduced hepatic IRI (Figure 2D). Moreover serum levels of the Cytokines TNF-α, IL-6 and IL-1β (Figure 3A–C), were marked reduced in UNC5B^+/−^ mice. The expression of mRNA analysed from injured liver tissue revealed that TNF-α expression was not significantly altered, whereas IL-6 was induced in the injured liver tissue of WT but not in UNC5B^+/−^ animals. Furthermore we found that CXCL1 and CXCL2 are induced in WT but not in UNC5B^+/−^ animals after IRI (Figure S1). This was accompanied by significant reduced infiltration of PMN into affected liver lobes (Figure 4A and C) in UNC5B^+/−^ mice and confirmed by reduced MPO activity measured from the injured liver tissue (Figure 4B).

### Functional inhibition of UNC5B is protective during hepatic ischemia-reperfusion injury

Finally we tested therapeutic potential of functional inhibition of UNC5B in WT mice by injection of a blocking anti-UNC5B antibody. Mice treated with the antibody showed significant reduced serum levels of LDH, ALT and AST as compared with control animals after hepatic IRI (Figure 5A–C). TTC staining of liver lobes further confirmed these results (Figure 5D). This was associated with significant reduced infiltration of PMN and MPO activity measured in injured liver tissues (Figure 6A–C). Additionally serum levels of the Cytokines TNF-α, IL-6 and IL-1β (Figure 7A–C) were lower in the antibody treated group without being significant.

## Discussion

A functional role of the NGP Receptor UNC5B was first described in the context of axonal guidance during CNS development. Recent data have however also shown a role for UNC5B in the immune system in particular during immune cell adhesion, migration and the orchestration of an acute inflammatory response. Since the pathophysiology of IRI underlies mechanisms of sterile inflammation [21] and UNC5B is implicated in these processes, it is not surprising that the UNC5B receptor is critically involved in renal IRI [17]. We therefore hypothesize that UNC5B might be of relevance during hepatic IRI. Thus we investigated the role of UNC5B during hepatic ischemia followed by reperfusion. We report here that UNC5B^+/−^ mice showed marked reduced hepatic IRI as measured by serum LDH, ALT and AST levels. This was associated with lower parameters of systemic inflammatory response. Additionally, the infiltration of neutrophils and their activity within the affected hepatic tissue was diminished in UNC5B^+/−^ mice. Moreover the pre-treatment of WT mice with an anti-UNC5B antibody significantly dampened liver injury, neutrophil infiltration and activity and trends to lower systemic cytokine levels. As such we provide evidence that UNC5B act as pro-inflammatory receptor during hepatic IRI and might serve as potential drug target to prevent hepatic IRI in the future.

In Vertebrates UNC5B mediates chemorepellant properties of the NGP netrin-1 during development of the CNS [22]. But UNC5B is not only located in the CNS. To confirm previous studies [9], [17] we found robust UNC5B expression within heart, lung, liver, intestine, spleen, blood, neutrophils, macrophages and lymphocytes. This expression pattern suggests that UNC5B holds function outside the CNS. And indeed UNC5B is implicated into different processes such as angio- [18] and cancerogenesis [23] as well as the control of acute inflammation [9] and IRI [17]. Loss of UNC5B gene expression during angiogenesis yield to extended endothelial tip cell filopodia, abnormal vessel branching and navigation [18]. During cancerogenesis UNC5B is downregulated in various kinds of tumor [23] affecting anchorage-independent growth and invasiveness and cell survival. Evidentially UNC5B as dependence receptor mediates p-53 induced apoptosis by its death domain [24] which is cleaved off when netrin-1 is not bound to the UNC5B receptor.

Since cell migration, adhesion and survival are critical characteristics influencing acute inflammatory processes it might be expected that UNC5B could be involved into the control of inflammation and IRI. Concerning leukocyte migration properties there is controversial data about the role of UNC5B. Recent studies employing the UNC5B ligand netrin-1 [9], [17] showed that netrin-1 inhibits migration and infiltration of affected tissues by immunocompetent cells like PMN and monocytes in UNC5B dependent manner. Treatment with an anti-UNC5B antibody abolished the effects of netrin-1 during fMLP-induced chemotaxis in vitro [9] and renal ischemia reperfusion in vivo [17]. Additionally Mirakaj et al. [25] showed that the anti-inflammatory effect of netrin-1 during Zymosan A induced Peritonitis is mediated by the A2BAR receptor. This is supported by data of Aherne et al. [26] who showed that UNC5B blockade was not able to affect DSS induced Colitis but was A2BAR receptor dependent. In contrast our data provides evidence that heterozygous knock out and functional inhibition of UNC5B reduces hepatic IRI. Reduced hepatic IRI was displayed by lowered serum LDH, ALT, AST, TTC staining and cytokine levels in the serum and the injured liver tissue, as well as reduced neutrophil infiltration and activity in the injured liver. Since LDH, ALT, AST are surrogate parameters for liver damage we only can speculate if our results are due to reduced tissue damage via diminished p-53 signalling or altered migration and adhesion properties of neutrophils. Furthermore, the crucial differences between our and other studies employing anti-UNC5B antibodies are the time-point, the way of the antibody administration and the dose used. We perfomed i.v. injection of the anti-UNC5B antibody 15 minutes prior to surgical procedures. The dose was 5 μg/mouse (200 µg/kg body weight). In the study of Tadagavadi et al. [17] the UNC5B blocking antibody was injected i.p. 18 hours before the induction of IRI in a dose of 800 µg/kg body weight. Aherne et al. [26] started the i.p. administration of the UNC5B blocking antibody 2 days before induction of DSS colitis. The dose of the UNC5B blocking antibody in this study was 800 µg/kg body weight. Therefore the differences in the findings from our study to these previously published data might be explained by the different time-point, way of administration and dose of the UNC5B blocking antibody.

Taken together, we report here that repression and functional inhibition of UNC5B is protective during the early phase of hepatic IRI holds protective potential and might be a strategy in the future to reduce tissue damage associated with reperfusion injury.

## Supporting Information

Figure S1 **UNC5B^+/−^ animals display altered cytokine expression after hepatic IRI.** RT-PCR was performed from liver samples. Relative mRNA expression of A) TNF-α, B) IL-6, C) CXCL1 and D) CXCL2 are shown. Data are mean ± SEM, n = 5 per group
(TIF)
