# Supplementary figures and images for: The Uncoordinated-5 Homolog B Receptor Affects Hepatic Ischemia Reperfusion Injury
Source: PLoS One. 2012 Jul 25;7(7):e41085. doi: 10.1371/journal.pone.0041085 (PMC3405071; doi:10.1371/journal.pone.0041085)

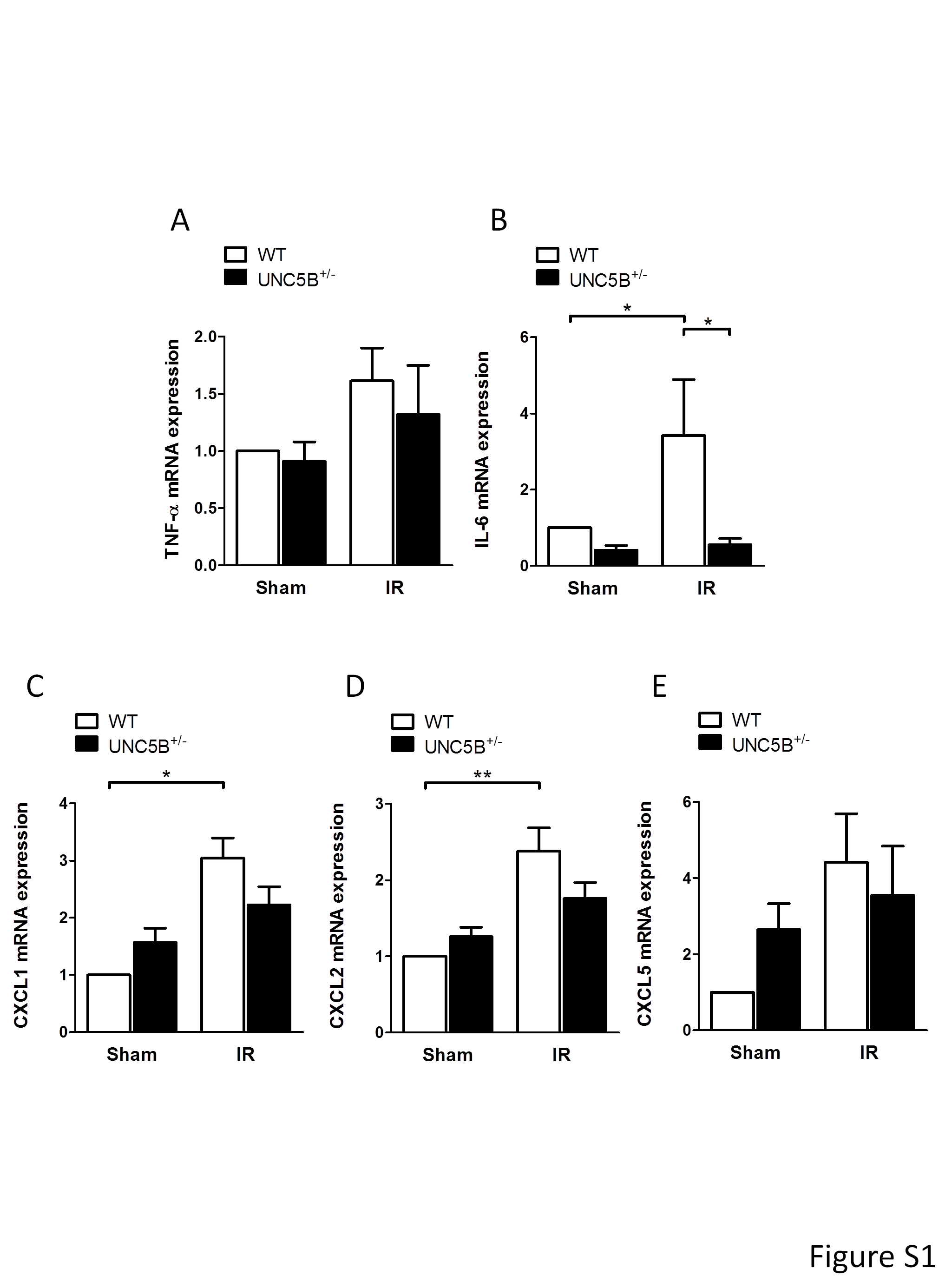

Supplement: Figure S1 — UNC5B+/− animals display altered cytokine expression after hepatic IRI. RT-PCR was performed from liver samples. Relative mRNA expression of A) TNF-α, B) IL-6, C) CXCL1 and D) CXCL2 are shown. Data are mean ± SEM, n = 5 per group (TIF) [file pone.0041085.s001.tif]
